# Osteopathic manipulative treatment for pandemic influenza: many questions, few answers

**DOI:** 10.1186/1750-4732-1-12

**Published:** 2007-07-09

**Authors:** Brent W Sanderlin, John C Licciardone

**Affiliations:** 1 Department of Family Medicine, University of North Texas Health Science Center-Texas College of Osteopathic Medicine, Fort Worth, TX 76107, USA; 2 Osteopathic Research Center, University of North Texas Health Science Center-Texas College of Osteopathic Medicine, Fort Worth, TX 76107, USA

## Abstract

Commentary on

Avian influenza: an osteopathic component to treatment

Raymond J Hruby and Keasha N Hoffman

In this volume, an article by Hruby and Hoffman titled "Avian influenza: an osteopathic component to treatment" proposes the use of osteopathic manipulative treatment (OMT) as an adjunct to other forms of therapy in the management of a potential pandemic of avian influenza or "bird flu" [1]. This article is certainly timely and presents a much-needed reminder of the potential use of OMT in the treatment of infectious diseases, especially those of the respiratory system.

The article does, however, raise several important questions. First, with regard to the evidence gathered during the 1918–1919 influenza pandemic, the authors cited a difference in survival between those patients treated by osteopathic and allopathic physicians [2]. They also acknowledged limitations of such retrospective studies. In our view, foremost among these is the difficulty in establishing a valid causal relationship between use of OMT and decreased mortality in an observational study. A crucial, but unknown, factor involves the comparability of patients seen by osteopathic and allopathic physicians during the 1918–1919 influenza pandemic. For example, were osteopathic patients less likely to have been residents of crowded urban areas than patients of allopathic physicians and, therefore, at lower risk of secondary infections and other environmental exposures that may have augmented influenza complications and mortality? Another unknown factor is whether OMT was the only difference in influenza treatment between osteopathic and allopathic physicians. If not, other factors may explain the reported differences in influenza outcomes. Other potential sources of selection bias and confounding may also have been at play, but they cannot be adequately assessed based on the limited methodological details provided in the cited report. Importantly, while Hruby and Hoffman also cite numerous other studies to support the use of OMT during an influenza pandemic, these studies are, methodologically, at the base of the evidence pyramid and generally provide only a theoretical basis for using OMT rather than evidence of its efficacy.

Second, with regard to the virus, we do not know what the potential infectivity, virulence, or mortality of a future strain of influenza might be, or even whether or not it would be the H5N1 strain currently making its way through the world bird populations. Because future mutations of the virus may alter infectivity, virulence, or mortality of the disease in either a positive or negative direction, predictions regarding a potential influenza pandemic are, at best, guesses. Most efforts to predict the potential effects on populations are based on the 1918–1919 influenza pandemic or smaller subsequent pandemics in later decades. Current computer models [3] based on the 1918–1919 influenza pandemic predict rapid and widespread transmission of the disease, affecting millions of persons in the United States alone. Disease incidence could be much greater not only because of our increased population density as compared with 1918–1919, but also because of the frequency of international travel in our society. Major metropolitan areas could be exposed to the disease simultaneously and the virus could revisit the population in multiple waves over the course of weeks to months. If these models are correct, such a pandemic could wreak havoc with both our current medical and mortuary systems, not to mention potential devastating effects on the world economy.

If a pandemic virus proves to be both highly infective and virulent, the potential pool of osteopathic physicians available to provide treatment to patients could shrink dramatically for two reasons. First, a decrement in available osteopathic physicians would be directly attributable to infection itself. Second, the osteopathic workforce could be indirectly diluted by public health responses that encourage people to "stay home" or that use social distancing methods such as closing schools and restricting major social events to limit spread of the disease. Given that the OMT techniques presented by Hruby and Hoffman can be time consuming and that the fewer available osteopathic physicians would be seeing many more patients than under normal circumstances, unless many other persons are trained in these techniques, OMT might not have a major impact in a pandemic. Certainly, if the disease were less widespread or less virulent, OMT potentially could be a useful adjunct to other therapies, and if the pandemic were severe enough it might prove to be one of few useful therapies available. Training allopathic physicians and even non-physicians in these OMT techniques would therefore become critical to their successful deployment in such a pandemic. Public health authorities on all levels also would be involved with this training and educated about the OMT techniques. However, developing awareness among and training programs for medical and public health professionals might prove to be a difficult undertaking in its own right.

Third, with regard to ambulatory medical care, there is little evidence to indicate that osteopathic physicians commonly use OMT in the treatment of respiratory diseases. We used data from the National Ambulatory Medical Care Surveys conducted in 2003 [4] and 2004 [5] to estimate the frequency of OMT provided in the treatment of influenza, pneumonia, or bronchitis throughout the United States. An estimated 3.0 million patient visits involved treatment of any of these three respiratory diseases by osteopathic physicians. Osteopathic manipulative treatment was reportedly used in no more than an estimated 35,000 (1%) of these patient visits. Clearly, the prevailing practice patterns among osteopathic physicians suggest a sizeable and perhaps insurmountable barrier in retooling to integrate the applicable OMT techniques in the event of an influenza pandemic.

While we applaud the efforts of Hruby and Hoffman to identify OMT techniques for pandemic influenza and related complications, as well as provide a manual for their use, considerable questions and doubt remain about efficacy and feasibility of implementation. Consequently, before proposing that OMT be used on a large scale during an influenza pandemic, we believe that more rigorous research, intensive planning, and coordinated medical and public health responses are warranted.
